# Pre-steady-state Kinetics Reveal the Substrate Specificity and Mechanism of Halide Oxidation of Truncated Human Peroxidasin 1*

**DOI:** 10.1074/jbc.M117.775213

**Published:** 2017-01-31

**Authors:** Martina Paumann-Page, Romy-Sophie Katz, Marzia Bellei, Irene Schwartz, Eva Edenhofer, Benjamin Sevcnikar, Monika Soudi, Stefan Hofbauer, Gianantonio Battistuzzi, Paul G. Furtmüller, Christian Obinger

**Affiliations:** From the ‡ Department of Chemistry, Division of Biochemistry, Vienna Institute of BioTechnology, BOKU-University of Natural Resources and Life Sciences, Muthgasse 18, A-1190 Vienna, Austria and; the Departments of § Life Sciences and; ¶ Chemistry and Geology, University of Modena and Reggio Emilia, 41125 Modena, Italy

**Keywords:** enzyme catalysis, extracellular matrix, heme, kinetics, peroxidase

## Abstract

Human peroxidasin 1 is a homotrimeric multidomain peroxidase that is secreted to the extracellular matrix. The heme enzyme was shown to release hypobromous acid that mediates the formation of specific covalent sulfilimine bonds to reinforce collagen IV in basement membranes. Maturation by proteolytic cleavage is known to activate the enzyme. Here, we present the first multimixing stopped-flow study on a fully functional truncated variant of human peroxidasin 1 comprising four immunoglobulin-like domains and the catalytically active peroxidase domain. The kinetic data unravel the so far unknown substrate specificity and mechanism of halide oxidation of human peroxidasin 1. The heme enzyme is shown to follow the halogenation cycle that is induced by the rapid H_2_O_2_-mediated oxidation of the ferric enzyme to the redox intermediate compound I. We demonstrate that chloride cannot act as a two-electron donor of compound I, whereas thiocyanate, iodide, and bromide efficiently restore the ferric resting state. We present all relevant apparent bimolecular rate constants, the spectral signatures of the redox intermediates, and the standard reduction potential of the Fe(III)/Fe(II) couple, and we demonstrate that the prosthetic heme group is post-translationally modified and cross-linked with the protein. These structural features provide the basis of human peroxidasin 1 to act as an effective generator of hypobromous acid, which mediates the formation of covalent cross-links in collagen IV.

## Introduction

Human peroxidasin 1 (hsPxd01)^3^ plays a critical role in the stabilization of basement membranes by catalyzing the formation of covalent cross-links within the collagen IV network (1). Type IV collagen α chains form triple helical protomers that self-assemble with end-to-end C-terminal associations known as NC1 hexamers. Peroxidasin 1 oxidizes bromide to hypobromous acid, which is responsible for the generation of a highly specific sulfilimine bond between opposing methionine and hydroxylysine residues that bridge the trimer-trimer interface of the NCI hexamer thereby structurally reinforcing the collagen IV network (2). Despite these very exciting findings in recent years, relatively little is known about the mechanism of the catalytic reactions, redox intermediates, and substrate specificity of this novel multidomain human heme peroxidase.

Human peroxidasin 1 belongs to the peroxidase-cyclooxygenase superfamily (3, 4). In addition to the catalytic peroxidase domain (POX), hsPxd01 comprises a leucine-rich repeat domain (LRR) and four C-like immunoglobulin domains (Ig) at the N terminus and a C-terminal von Willebrand factor type C module (VWC), all known to be important for protein-protein interactions and cell adhesion. Moreover, mature human peroxidasin 1 is shown to be highly glycosylated and to form a homotrimer via intermolecular disulfide bonds (5, 6).

The peroxidase domain displays high homology to that of the well characterized chordata peroxidases lactoperoxidase (LPO), myeloperoxidase (MPO), eosinophil peroxidase (EPO), and thyroid peroxidase (TPO) with the highest similarity to LPO. Comparative sequence analysis clearly suggests that in the active site of hsPxd01 all amino acid residues, which are crucial to peroxidase and halogenation activity, are fully conserved (5, 7), including distal His, Arg, and Gln.

It has been reported that the catalytic efficiency of bromide oxidation (*k*_cat_/*K_m_*) of recombinant full-length hsPxd01 is rather low but increased upon truncation (5). This was confirmed in a recent study that showed the cleavage of trimeric hsPxd01 at Arg^1336^ C-terminal of the peroxidase domain by a proprotein convertase (8). The truncation eliminates the von Willebrand factor and renders the peroxidase more active. This proteolytic maturation seems to represent a key regulatory event in hsPxd01 biosynthesis and function because the C-terminal proprotein convertase recognition sequence is evolutionarily conserved throughout the animal kingdom (8).

**FIGURE 1.**
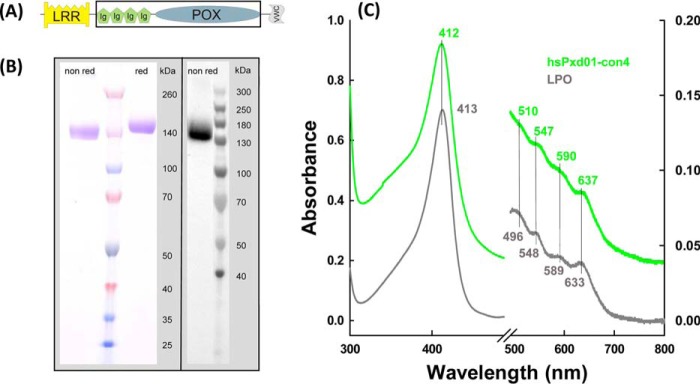
**Biochemical characterization of hsPxd01-con4.** *A,* schematic structure of hsPxd01-con4. It includes four Ig domains (*Ig*) and the catalytic POX domain as marked by the *black box*, omitting the LRR domain and the VWC module of full-length hsPxd01. *B,* SDS-PAGE and detection of covalently bound heme by ECL of hsPxd01-con4: 2 μg of protein was resolved on a 4–12% gradient gel under non-reducing and under reducing conditions (*left panel*). For ECL 6 μg of hsPxd01-con4 was blotted on a nitrocellulose membrane, and covalently bound heme was visualized with enhanced chemiluminescence (*right panel*). *C,* UV-visible spectra of hsPxd01-con4 and bovine lactoperoxidase: spectra of 8 μm per heme hsPxd01-con4 (*green*) and bovine lactoperoxidase (*bLPO*) (*gray*) were recorded in 100 mm phosphate buffer, pH 7.4.

In this work, to study the reactivity of the peroxidase domain and the effect of other domains on catalysis, several truncated variants of hsPxd01 were expressed recombinantly in HEK cells (5). One monomeric construct composed of the four Ig domains and the peroxidase domain (hsPxd01-con4) (Fig. 1*A*) was superior in terms of yield, heme insertion, spectroscopic features, and catalytic activity. This allowed for the first time a comprehensive study of the kinetics of interconversion of the relevant redox intermediates of the halogenation cycle. Here, we report the standard reduction potential of the Fe(III)/Fe(II) couple of the peroxidase domain and apparent bimolecular rate constants for cyanide binding as well as the formation and reduction of compound I mediated by hydrogen peroxide, bromide, iodide, and thiocyanate at pH 7.4. Based on the available structural, kinetic, and thermodynamic data of the homologous human peroxidases, we provide a mechanism for the halogenation cycle of human peroxidasin 1, and we discuss its relevance for its biosynthetic function in collagen IV cross-linking.

## Results

### 

#### 

##### Purification, Spectral and Redox Properties of hsPxd01-con4

Transient expression of hsPxd01-con4 in HEK cells resulted in a yield of purified protein of ∼10–20 mg/liter medium. The protein was composed of the amino acid residues Pro^246^–Asp^1314^ (numbers referring to full-length hsPxd01, including the signal peptide (5)) with a theoretical molar mass of 121 kDa. The construct included the four immunoglobulin (Ig)-like domains and the POX domain. In SDS-PAGE under both aerobic and reducing conditions, the corresponding band appeared at a slightly higher molar mass (Fig. 1*B*, *left panel*) because hsPxd01 is highly glycosylated with eight confirmed *N*-glycosylation sites located in the hsPxd01-con4 region (5). The heme of this construct was post-translationally modified and covalently bound to the protein that was clearly demonstrated by SDS-PAGE in combination with the enhanced chemiluminescence staining procedure (Fig. 1*B*, *right panel*). Covalent attachment of the prosthetic group could also be confirmed by precipitation of the recombinant protein by acetone at pH 4.5 resulting in colorless supernatants (data not shown). Furthermore, the determined spectral and redox properties clearly suggested the establishment of heme-protein bonds (see below). It is well known that these post-translational modifications considerably influence both the spectral signatures of a heme protein in its ferric state and the standard reduction potential of the Fe(III)/Fe(II) couple (9, 10).

In this context, it is important to note that the purification protocol (see under “Experimental Procedures”) could be significantly improved by including a 48-h incubation step prior to buffer exchange and affinity chromatography. This period seemed to be necessary for establishment of covalent bonds. The modification of the prosthetic group is reflected by a gradual transition of the UV-visible spectrum of freshly purified hsPxd01-con4. Spectral transition included a red-shift of the Soret band from 410 to 412 nm together with establishment of Q-bands at 510, 547, and 590 nm and a charge transfer (CT) band at 637 nm (Fig. 1*C*, *green spectrum*). This spectrum is reminiscent of that of ferric LPO (Fig. 1*C*, *gray spectrum*). The peroxidase domain of hsPxd01 has a high amino acid sequence homology with LPO (34% identity and 53% similarity) (5). In the known crystal structure of LPO, continuous electron densities underline the presence of two ester bonds between the modified prosthetic heme group and conserved Asp and Glu residues (7). Sequence alignment and modeling demonstrated that these acidic amino acids are fully conserved in hsPxd01 (*i.e.* Asp^826^ and Glu^980^) (3, 5).

**FIGURE 2.**
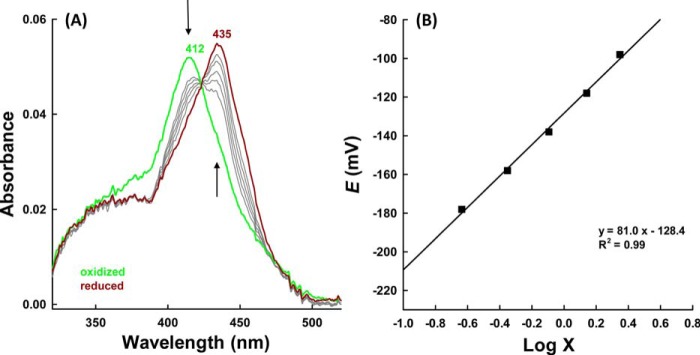
**Spectroelectrochemistry of hsPxd01-con4.** *A,* electronic spectra of hsPxd01-con4 recorded at various potentials in spectroelectrochemical experiments carried out with an OTTLE cell at 25 °C. Conditions: 5 μm hsPxd01-con4 in 100 mm phosphate buffer, pH 7.4, containing 100 mm NaCl, in the presence of the following mediators: 30 μm methyl viologen, 1 μm lumiflavin 3-acetate; methylene blue, phenazine methosulfate; and indigo disulfonate used as mediators. *Arrows* indicate spectral changes during reduction from ferric (*green*) to ferrous hsPxd01-con4 (*brown*). *B,* corresponding Nernst plot where × represents (*A*_λred_^max^ − *A*_λred_)/(*A*_λox_^max^ − *A*_λox_), with *A*_λox_ = 412 nm and *A*_λred_ = 435 nm, respectively.

Heme modification was also underlined by spectroelectrochemical studies on hsPxd01-con4. Fig. 2 shows a representative redox titration with fully oxidized and fully reduced hsPxd01-con4 depicted in *green* and *brown*, respectively. Upon reduction, the Soret peak of the ferric protein shifted from 412 to 435 nm (Fig. 2) very similar to 5-coordinated ferrous lactoperoxidase and eosinophil peroxidase (11). From the corresponding Nernst plot (Fig. 2*B*), the standard reduction potential (*E*′^0^) of the Fe(III)/Fe(II) couple was calculated to be −0.128 ± 0.006 V (25 °C and pH 7.4).

##### Cyanide Binding

**FIGURE 3.**
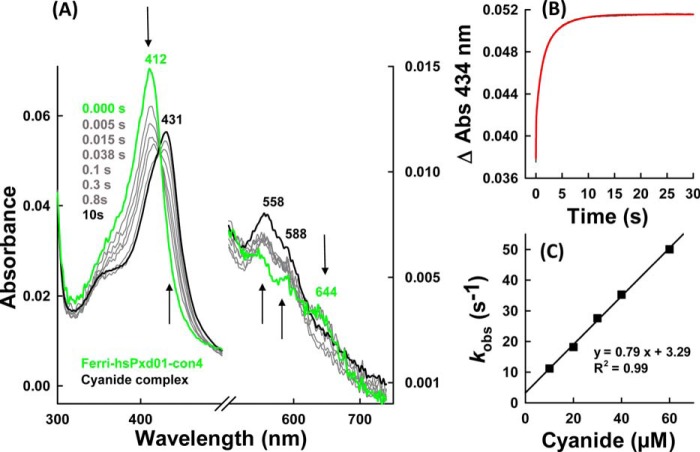
**Cyanide binding by ferric hsPxd01-con4.** *A,* spectral changes (*arrows*) of 1 μm hsPxd01-con4 upon addition of 1 mm sodium cyanide in 100 mm phosphate buffer, pH 7.4. *B,* time trace and fit (*red*) of 500 nm hsPxd01-con4 after adding 90 μm cyanide in 100 mm phosphate buffer, pH 7.4. Spectral changes were recorded at 434 nm. *C, k*_obs_ values for the reaction of 500 nm hsPxd01-con4 reacting with 10–60 μm cyanide in 100 mm phosphate buffer, pH 7.4, plotted against the cyanide concentration for determination of *k*_on_, *k*_off_, and *K_D_*.

Before investigating the kinetics of compound I formation and reduction, we probed the accessibility of the heme cavity and the homogeneity of the architecture of the active site by monitoring the kinetics of cyanide binding. It has been demonstrated that the post-translational modification of the prosthetic group in peroxidases from the heme peroxidase-cyclooxygenase superfamily is often not fully established resulting in some structural heterogeneity of the heme cavity that can easily be probed by studying the kinetics of cyanide binding (7, 10). In the case of hsPxd01-con4, cyanide binding resulted in the transition of the high spin (*S* = 5/2) Fe(III) state to the low spin (*S* = 1/2) Fe(III) state (Soret maximum at 431 nm, bands at 558 and 588 nm, and loss of the CT band at 637 nm) with clear isosbestic points at 423, 494, 519, 623, and 666 nm (Fig. 3*A*). Binding of the ligand was biphasic with a dominating rapid first phase and a slower second phase (Fig. 3*B*). From the double exponential fit of these time traces, first-order rate constants (*k*_obs(1)_ and *k*_obs(2)_) were obtained and plotted *versus* cyanide concentration. From the corresponding linear plots (Fig. 3*C*), an apparent second-order rate constant *k*_on(1)_ for the dominating rapid phase was calculated to be (7.9 ± 0.2) × 10^5^ m^−1^ s^−1^ (*k*_off_ = (3.3 ± 0.7) s^−1^ and *K_D_*(1) = *k*_off_/*k*_on_ = 4.2 μm) at pH 7.4 and 25 °C, which compares with 1.3 × 10^6^ m^−1^ s^−1^ and *K_D_* = 4.3 μm for human MPO (12) and 1.3 × 10^6^ m^−1^ s^−1^ and *K_D_* = 23.8 μm for bovine LPO (13). From the plot of *k*_obs(2)_ values *versus* cyanide concentration, an apparent *k*_on(2)_ of (8.8 ± 0.2) × 10^3^ m^−1^ s^−1^ (*k*_off(2)_ = (0.40 ± 0.08) s^−1^ and a *K_D_*(2) = 45.5 μm) (data not shown) was calculated for the minor second phase. These binding parameters are comparable with recently reported values for a recombinant bacterial peroxidase from the cyanobacterium *Lyngbya* sp. PCC 8106, which features autocatalytically formed covalent heme to protein links (10). Like the peroxidase domain of hsPxd01, the bacterial peroxidase has a high amino acid sequence homology with LPO, and the prosthetic group is covalently attached to the protein via two ester bonds. The reaction of the ferric bacterial protein with cyanide also showed a biphasic behavior with a dominating rapid phase. In both cases in a small portion of the protein the post-translational modification of the prosthetic group was not fully accomplished resulting in some heterogeneity of the active site and in consequence in the kinetics and thermodynamics of cyanide binding.

##### Hydrogen Peroxide Efficiently Oxidizes hsPxd01-con4 to Compound I

**FIGURE 4.**
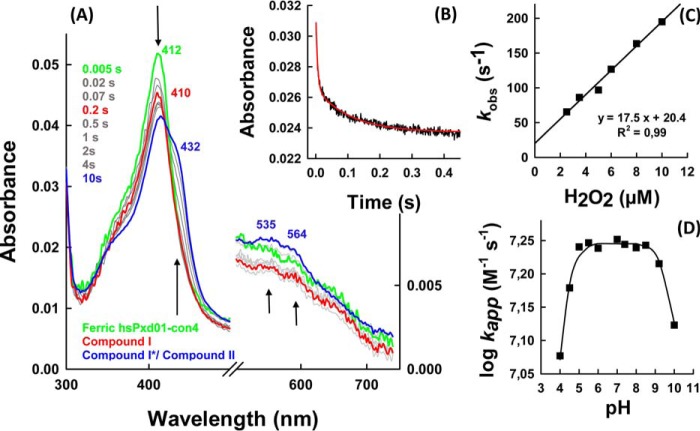
**Formation of compound I.** *A,* spectral changes (*arrows*) of 1 μm hsPxd01-con4 reacting with equimolar hydrogen peroxide in 100 mm phosphate buffer, pH 7.4. *B,* typical time trace of compound I formation followed at 412 nm (*black line*) with corresponding double exponential fit (*red line*). *C,* determination of the apparent second-order rate constant at 25 °C: 1 μm hsPxd01-con4 was reacted with 2.5, 3.5, 5, 6, 8, and 10 μm hydrogen peroxide, and the obtained pseudo-first order *k*_obs_ values of the first phase were plotted against the concentration. *D,* pH profile of hsPxd01-con4 compound I formation: the determined *k*_app_ values at pH 4, 4.5, 5, 5.5, 6, 7, 7.4, and 8–10 were plotted against the respective pH values.

To act as peroxidase in extracellular cross-linking reactions, peroxidasin must be oxidized by peroxides. Here, we showed that hydrogen peroxide efficiently converted the ferric form of hsPxd01-con4 into the redox intermediate compound I. Fig. 4*A* shows that this reaction was reflected by a spectral transition with isosbestic points at 360 and 443 nm, hypochromicity in the Soret absorbance (maximum at 410 nm), and the establishment of a new band around 670 nm (Fig. 4, *red spectrum*). Similar to LPO (14), maximum hypochromicity was already achieved with equimolar H_2_O_2_ within 200 ms (Fig. 4*A*). Analogous to LPO (14), compound I of hsPxd01-con4 was not stable but slowly converted to a compound II-like species (Fig. 4, *blue spectrum*).

The kinetics of hsPxd01-con4 oxidation mediated by H_2_O_2_ was followed by the decrease of absorbance at 412 nm. The reaction was biphasic with a dominating rapid first phase (>80% of Δ*A*_412 nm_). The corresponding pseudo first-order rate constants *k*_obs(1)_ and *k*_obs(2)_ were obtained from double exponential fits. From the slope of the plots of the respective *k*_obs_ values *versus* hydrogen peroxide concentration, the apparent bimolecular rate constants for the dominating phase *k*_app(1)_, (1.8 ± 0.1) × 10^7^ m^−1^ s^−1^ (Fig. 4*C*), and the second phase *k*_app(2)_, (6.9 ± 0.6) × 10^5^ m^−1^ s^−1^ (data not shown), were calculated, pH 7.4.

Furthermore, we could demonstrate that the rate of compound I formation was invariant within the pH range of 5.0–9.0 (Fig. 4*D*). A p*K*_1_ value of 4.7 and a p*K*_2_ value of 9.0 were calculated from the fit *k*_app_ = *a*/(1 + *x*/*b*) × (1 + *c*/*x*) with *a* representing *k*_internal_; *x* is the concentration of H^+^; *b* is p*K*_1_, and *c* is p*K*_2_. The first inflection point might reflect the p*K_a_* of the distal histidine that acts as proton acceptor in compound I formation of heme peroxidases (7), whereas the second one could be related with the alkaline transition of hsPxd01-con4. The UV-visible spectrum of the ferric high spin protein is invariant between pH 5 and 8.0 (5) but converts to a low spin spectrum with red-shifted Soret band (430 nm) at pH values >8.5 most probably reflecting the formation of a low spin hydroxide complex.

**FIGURE 5.**
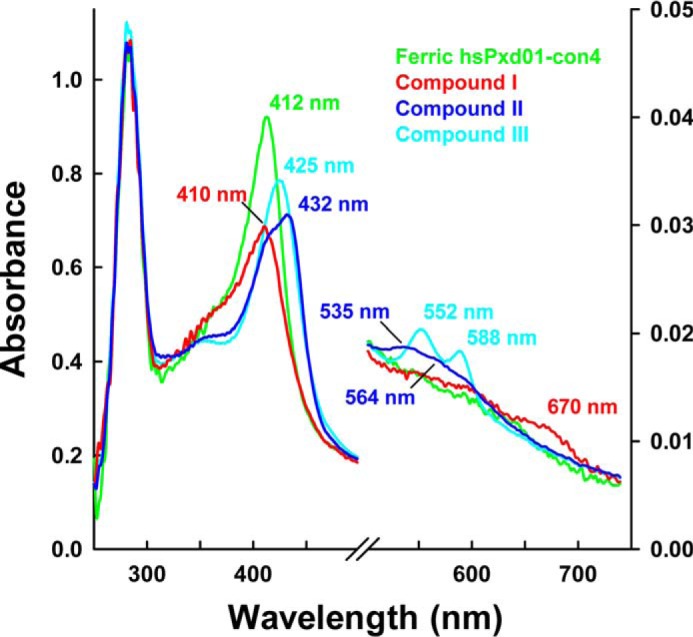
**UV-visible spectra of compound I, compound II, and compound III.** 1 μm hsPxd01-con4 was reacted with varying concentrations of hydrogen peroxide in 100 mm phosphate buffer, pH 7.4. The ferric form of hsPxd01-con4 is depicted in *green*, and compound I, compound II, and compound III are shown in *red, blue,* and *cyan*, respectively. The characteristic Soret peak maxima and bands in the visible region are illustrated in the same color code. Compound II was formed by the addition of 50 μm hydrogen peroxide, and compound III was generated by adding 1 mm hydrogen peroxide to the ferric protein.

Addition of excess hydrogen peroxide to ferric hsPxd01-con4 converts the enzyme from the ferric state via compound I to compound II (oxoiron(IV) species) and, finally, to compound III, which resembles electronically oxyhemoglobin or oxymyoglobin (*i.e.* Fe(II)-O_2_ ↔ Fe(III)-O_2_^˙̄^) (Fig. 5). When hsPxd01-con4 was mixed with a 50-fold molar excess of hydrogen peroxide, the formation of predominantly compound II was observed with a heme Soret maximum at 432 nm and a broad Q band at 535 nm with a shoulder at 564 nm (Fig. 5, *blue spectrum*). With a 1000-fold molar excess of H_2_O_2_, compound II was converted to compound III resulting in a distinct UV-visible spectrum with a heme Soret maximum at 425 nm and prominent Q-bands at 552 and 588 nm (Fig. 5, *cyan spectrum*).

**FIGURE 6.**
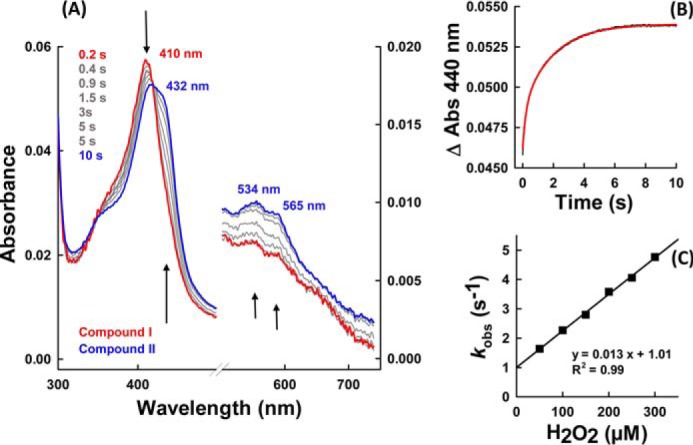
**Reduction of compound I to compound II mediated by hydrogen peroxide.** *A,* spectral changes (*arrows*) of 1 μm hsPxd01-con4 compound I reacting with 50 μm hydrogen peroxide. Compound I was formed after 200 ms in the aging loop. *B,* time trace of reaction between 500 nm compound I of hsPxd01-con4 and 300 μm hydrogen peroxide measured in the sequential stopped-flow mode (delay time of 200 ms for compound I formation). The time trace (*black line*) was fitted double exponentially (*red line*). *C,* pseudo first-order rate constant of 500 nm hsPxd01-con4 compound I reacting with 50, 100, 150, 200, 250, and 300 μm hydrogen peroxide, respectively. *k*_obs_ values of the first phase were plotted against the concentration of hydrogen peroxide.

It was interesting to see that in contrast to LPO (14) but similar to myeloperoxidase (7), the conversion of compound I to compound II was also dependent on the hydrogen peroxide concentration (Fig. 6). After incubating hsPxd01-con4 with equimolar H_2_O_2_ in the aging loop of the stopped-flow instrument for 200 ms, formed compound I was mixed with increasing concentrations of H_2_O_2_. Formation of compound II clearly depended on the H_2_O_2_ concentration, and from the biphasic time traces (Fig. 6*B*), *k*_app_ of the dominating initial reaction was calculated to be (1.3 ± 0.03) × 10^4^ m^−1^ s^−1^ (Fig. 6*C*).

##### Reaction of hsPxd01-con4-Compound I with Halides and Thiocyanate

**FIGURE 7.**
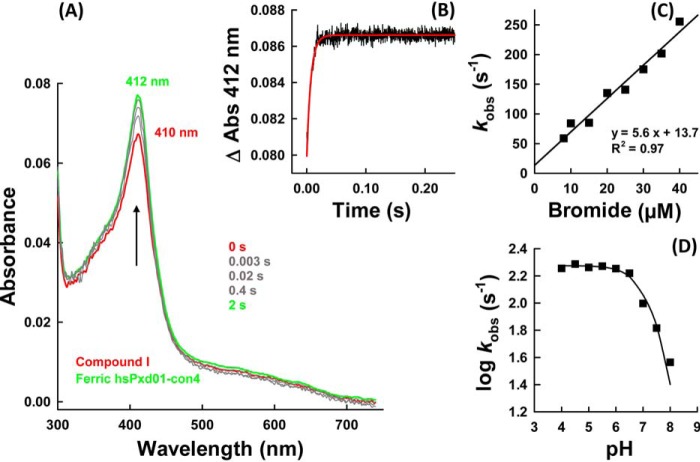
**Reaction of compound I with bromide.** *A,* spectral changes of the reaction between 1 μm compound I of hsPxd01-con4 (*red spectrum*) with 10 μm bromide in 100 mm phosphate buffer, pH 7.4. Compound I was formed with equimolar H_2_O_2_ in the sequential mode (delay time of 200 ms). *B,* time trace (*black line*) of reaction between 1 μm compound I of hsPxd01-con4 and 20 μm bromide in 100 mm phosphate buffer, pH 7.4, together with single exponential fit (*red line*). *C,* plot of *k*_obs_ values *versus* bromide concentration. Conditions: 1 μm hsPxd01-con4 compound I reacting with 8, 10, 15, 20, 25, 30, 35, and 40 μm bromide in 100 mm phosphate buffer, pH 7.4, respectively. *D,* pH dependence of bromide oxidation by compound I. Plot of log *k*_obs_ values *versus* pH. Conditions: 1 μm hsPxd01-con4 reacting with 10 μm bromide in the respective 100 mm buffer (pH 4–5.5 citrate phosphate buffer; pH 5.5–8 phosphate buffer; and pH 9 and 10 carbonate buffer).

Next, we probed the reactivity of hsPxd01-con4 compound I with the halides chloride, bromide, iodide, and the pseudo-halide thiocyanate. Again, the sequential mode was used to form compound I by preincubating hsPxd01-con4 with an equimolar concentration of hydrogen peroxide for 200 ms before the halides were added. Fig. 7*A* shows the direct conversion of compound I back to the ferric enzyme upon addition of bromide. In contrast to the reaction of the ferric enzyme with cyanide and hydrogen peroxide, compound I reduction by bromide was monophasic (Fig. 7*B*) and allowed the calculation of an apparent bimolecular rate *k*_app_ = (5.6 ± 0.4) × 10^6^ m^−1^ s^−1^ at pH 7.4 (Fig. 7*C*).

Because hypobromous acid formation was reported to be essential for the formation of the sulfilimine link (1, 2), the pH dependence of the kinetics of bromide oxidation was investigated. Fig. 7*D* depicts the corresponding plot of the logarithm of first-order rate constants *k*_obs_ *versus* pH. Between pH 4 and pH 6, the reaction was fast and pH-independent but decreased with increasing pH.

Importantly, chloride cannot act as an electron donor for compound I of hsPxd01-con4. Even in the presence of chloride concentrations ≫10 mm added to 1 μm hsPxd01-con4 compound I, no formation of the ferric enzyme could be detected. In the presence of chloride, compound I slowly converted to an intermediate with a compound II-like spectrum (data not shown). However, upon addition of 5 μm Br^−^ to 100 mm Cl^−^, the direct reduction of compound I to ferric hsPxd01-con4 could be observed (data not shown).

**FIGURE 8.**
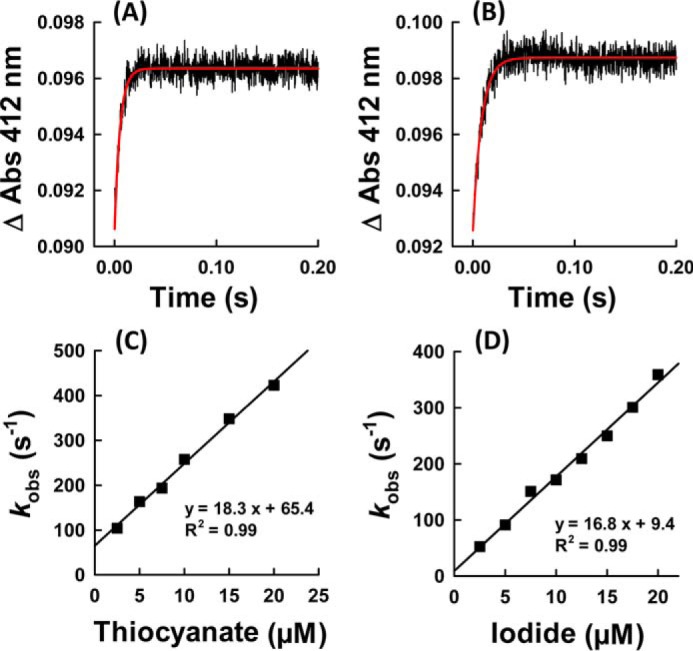
**Reaction of compound I with thiocyanate and iodide.** Time traces at 412 nm of reaction between 1 μm compound I of hsPxd01-con4 and 20 μm thiocyanate (*A*) and 5 μm iodide (*B*), respectively. Plots of *k*_obs_ values *versus* thiocyanate (*C*) and iodide (*D*) concentration. Conditions: 1 μm hsPxd01-con4 compound I reacting with 2.5, 5, 7.5, 10, 15, and 20 μm thiocyanate or 2.5, 5, 7.5, 10, 12.5, 15, 17.5, and 20 μm iodide in 100 mm phosphate buffer, pH 7.4.

By contrast, the reaction of hsPxd01-con4 compound I with thiocyanate and iodide was very fast and resulted in a direct conversion of compound I to the ferric state. The spectral transition was almost identical to that observed with bromide. Fig. 8, *A* and *B,* shows representative monophasic time traces that could be fitted single exponentially. The apparent bimolecular rate constants were calculated to be (1.8 ± 0.07) × 10^7^ m^−1^ s^−1^ and (1.7 ± 0.067) × 10^7^ m^−1^ s^−1^ for the reaction with thiocyanate and iodide at pH 7.4 (Fig. 8, *C* and *D*).

## Discussion

For the first time, a truncated variant of hsPxd01 was produced in appreciable yield, good quality, and high activity, which allowed for pre-steady-state kinetic measurements to evaluate the substrate specificity and the mechanism of halide oxidation of human peroxidasin 1. So far, only steady-state (5) and end point measurements (1, 2, 5) were published, and it was suggested that the enzyme preferentially generates hypobromous acid as a reactive intermediate to form sulfilimine cross-links in collagen (1, 2). Additionally, some papers reported the generation of hypochlorous acid by hsPxd01 (15, 16).

Human peroxidasin 1 is a homotrimeric, highly glycosylated multidomain peroxidase, which so far could only be produced in recombinant form in animal cell cultures in very low amounts of protein with unsatisfactory heme occupancy and incomplete post-translational heme modification and thus low activity (5). However, elimination of the LRR and VWC domains increased the activity of the respective recombinant construct (5). Recently, Colon and Bhave (8) demonstrated that proprotein convertase processing enhances peroxidasin 1 activity by elimination of the VWC domains and proposed that this event represents a key step in the biosynthesis and function of hsPxd01 to support basic membrane and tissue integrity. These findings motivated us to design several constructs, including the POX domain only to search for a functional and well folded protein for first comprehensive pre-steady-state kinetic studies. Finally, comparative biochemical studies demonstrated that only the construct hsPxd01-con4 fulfilled the requirements with regard to yield, heme occupancy, and modification. Apparently, the four Ig domains together with the peroxidase domain are the smallest active entity, which is also supported by data from Ero-Tolliver *et al.* (17) that showed that this construct is likewise the smallest unit that mediates efficient sulfilimine cross-linking. The POX domain only was inactive in these studies, which underlines the evolutionarily conserved function of peroxidasin in tissue development and integrity and distinguishes peroxidasin from other peroxidases, such as LPO, EPO, and MPO, which are composed of fully functional POX domains only.

Our spectral, redox, and kinetic data clearly demonstrate that hsPxd01-con4 has an LPO-like heme environment that was already proposed by sequence alignment and homology modeling (4, 5). It has been demonstrated that one of the most important structural features of halogenating enzymes like LPO, EPO, and MPO is the modification of the 1- and 5-methyl groups on pyrrole rings A and C of the heme group allowing formation of ester linkages with the carboxyl groups of conserved aspartate and glutamate residues (7, 18). Myeloperoxidase is unique in having a third covalent (*i.e.* sulfonium ion) bond (9, 19, 20). Formation of these covalent heme-protein bonds has been proposed to occur autocatalytically (22–24) mediated by (sub)micromolar hydrogen peroxide concentrations and has a deep impact on the biochemical and biophysical properties of these peroxidases (18, 19). Full establishment of the covalent bonds is never achieved even when native proteins are purified from natural sources (20, 22–24). Similarly, in the case of recombinantly produced members from this superfamily, there was always some heterogeneity that could be diminished to some extent by adding low micromolar amounts of H_2_O_2_ (10, 19, 25–27). In human peroxidasin 1 Asp^826^ and Glu^890^ have been proposed to be involved in heme-protein ester bonds (3–5). Freshly purified hsPxd01-con4 showed a red-shifted (compared with unmodified heme b) Soret maximum at 410 nm and a standard reduction potential of the Fe(III)/Fe(II) couple of −0.215 V (5), which already indicated the presence of partially modified heme b. But importantly, simply by keeping the protein under aerobic condition for 48 h prior to purification (see below), the Soret maximum further shifted to 412 nm (Fig. 1), and *E*′^0^ increased to −0.128 V (Fig. 2). Nevertheless, the biphasic behavior of cyanide binding to ferric hsPxd01-con4 (Fig. 3) or compound I formation (Fig. 4) indicated that there is still some heterogeneity left. Considering comparable data about homologous human peroxidases (10, 19, 25–27), it can be speculated that the observed heterogeneity of hsPxd01-con4 also derives from a mixture of molecules with mainly two ester linkages and a small portion having only one covalent bond. It has to be mentioned that this phenomenon is even observed in crystal structures of MPO (20) and LPO (28), which always show fully established Asp ester linkage but split electron densities for the Glu ester bond suggesting the presence of two conformations.

In any case, we could demonstrate that ferric hsPxd01-con4 exhibits spectral features very similar to LPO and EPO and in addition shows similar rates of cyanide binding, which clearly suggests comparable active site architectures. The standard reduction potential *E*′^0^ [Fe(III)/Fe(II)] of our construct follows the hierarchy *E*′^0^ (MPO; + 5 mV) > *E*′^0^ (hsPxd01-con4; −128 mV) > *E*′^0^ (EPO; −176 mV) > *E*′^0^ (LPO; −183 mV) (Table 1) (9, 11, 29).

**TABLE 1:**
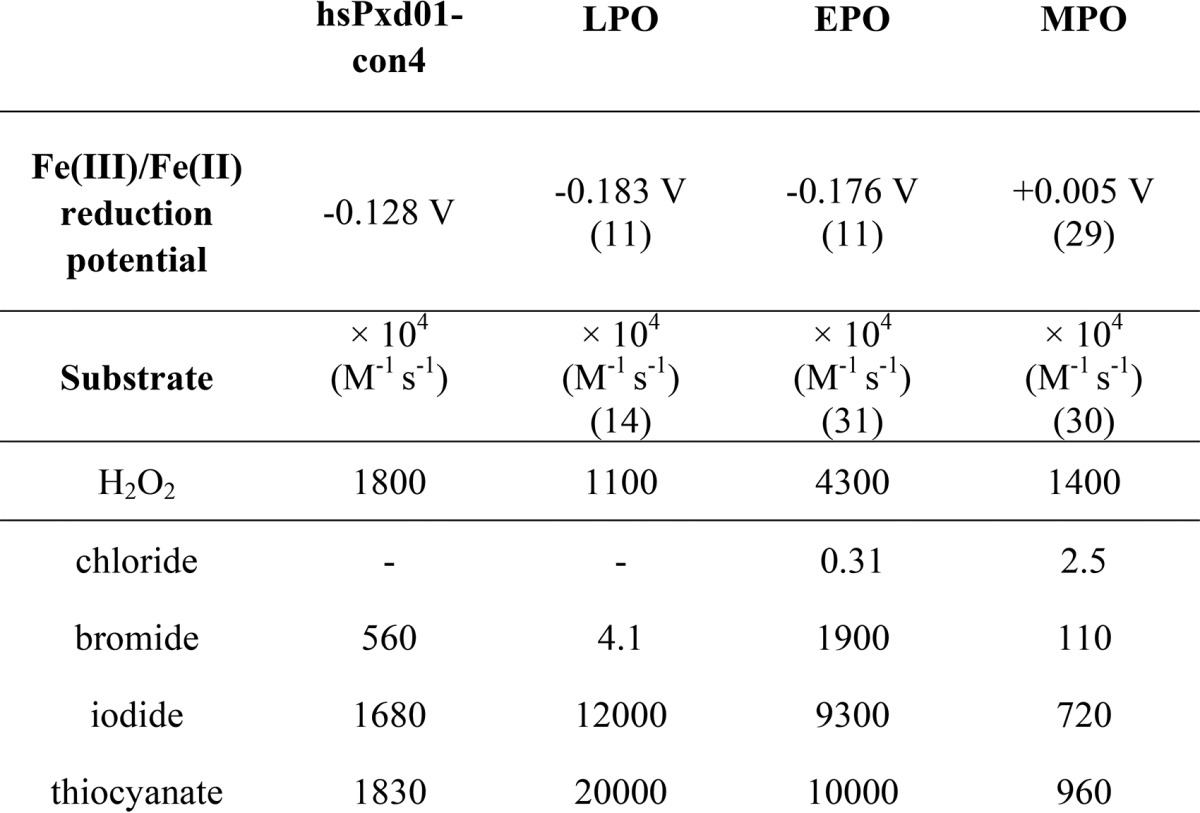
**Fe(III)/Fe(II) reduction potential and apparent second-order rate constants of Compound I formation (H_2_O_2_) and reduction (chloride, bromide, thiocyanate, and iodide) reactions of hsPxd01-con4, LPO, EPO and MPO** For hsPxd01-con4, all measurements were performed in 100mm phosphate buffer, pH 7.4,whereas the data displayed for LPO, EPO, and MPO was measured in 10 mm phosphate buffer, pH 7.

The reaction cycle of hsPxd01 starts by reaction of the Fe(III) form with hydrogen peroxide to form compound I (oxoiron(IV) with porphyrin π-cation radical), which contains two oxidizing equivalents more than the resting enzyme (Reaction 1). The determined *k*_app_ value of this bimolecular reaction was similar to that of other mammalian heme peroxidases with reported *k*_app_ values within (1.1–5.6) × 10^7^ m^−1^ s^−1^ (14, 30, 31). Heterolytic cleavage of hydrogen peroxide is supported by a fully conserved distal His-Arg pair (His^827^ and Arg^977^ in hsPxd01) (30), with His^827^ acting as proton acceptor and donor in this redox reaction. Upon its protonation (p*K_a_* ∼4.7) Reaction 1 cannot take place. PorFe is equal to heme or protoporphyrin IX plus iron.

$$\begin{array}{c} \left[\text{Por}\;\text{Fe}\left(\text{III}\right)\right]+{\text{H}}_{2}{\text{O}}_{2}\to \left[\text{Por}\overset{+}{\cdot }\text{Fe}\left(\text{IV}\right)=\text{O}\right]{\text{H}}_{2}\text{O}\\ \text{REACTION}\;1 \end{array}$$

Similar to LPO (14) and thyroid peroxidase (TPO) (32), compound I can be produced with equimolar H_2_O_2_. In the absence of an exogenous electron donor, it slowly converts to a compound II-like species, which most probably is compound I* formed by intramolecular electron transport from the protein matrix (where aa is amino acid) to the prosthetic group (Reaction 2).

$$\begin{array}{c} \left[\text{Por}\overset{+}{\cdot }\text{Fe}\left(\text{IV}\right)=\text{O}\;\text{aa}\right]\to \left[\text{Por}\;\text{Fe}\left(\text{IV}\right)-\text{OH}\;\text{aa}\overset{+}{\cdot }\right]\\ \text{REACTION}\;2 \end{array}$$

Interestingly, hydrogen peroxide also mediates the one-electron reduction of compound I of hsPxd01-con4 to compound II, *i.e.* [Por Fe(IV)-OH] (Reaction 3),

$$\begin{array}{c} \left[\text{Por}\overset{+}{\cdot }\text{Fe}\left(\text{IV}\right)=\text{O}\right]+{\text{H}}_{2}{\text{O}}_{2}\to \left[\text{Por}\;\text{Fe}\left(\text{IV}\right)-\text{OH}\right]+{\text{O}}_{2}\overset{-}{\cdot }{\text{H}}^{+}\\ \text{REACTION}\;3 \end{array}$$

which so far has been described for MPO only (30). At high (>1000) molar excess of H_2_O_2_, compound II is converted to compound III (Reaction 4).

$$\begin{array}{c} \left[\text{Por}\;\text{Fe}\left(\text{IV}\right)-\text{OH}\right]+{\text{H}}_{2}{\text{O}}_{2}\to {\text{H}}_{2}\text{O+}\left[\text{Por}\;\text{Fe}\left(\text{II}\right)-{\text{O}}_{2}\right]↔[\text{Por}\;\text{Fe}\left(\text{III}\right)-{\text{O}}_{2}\overset{-}{\cdot }\\ \text{REACTION}\;4 \end{array}$$

However, it is unlikely that this reaction is relevant *in vivo* because the extracellular H_2_O_2_ concentration is typically in the low micromolar range. Moreover, it is unknown whether there is a distinct (inducible?) source of hydrogen peroxide in the extracellular matrix for initiation of Reaction 1 and, finally, the halogenation cycle for sulfilimine formation.

It has been reported that the pseudohalide thiocyanate (SCN^−^) and iodide inhibited sulfilimine cross-linking in cell culture, whereas bromide enhanced cross-link formation (2). Now with our stopped-flow data, we can easily explain these observations. Direct reduction of compound I by halides (X^−^) or SCN^−^ restores the enzyme in its resting state and releases hypohalous acids (HOX) or hypothiocyanite (HOSCN) (Reaction 5).

$$\begin{array}{c} \left[\text{Por}\overset{+}{\cdot }\text{Fe}\left(\text{IV}\right)=\text{O}\right]+{\text{X}}^{-}+{\text{H}}^{+}\to \left[\text{Por}\;\text{Fe}\left(\text{III}\right)\right]+\text{HOX}\\ \text{REACTION}\;5 \end{array}$$

At physiological pH 7.4 both thiocyanate (1.83 × 10^7^ m^−1^ s^−1^) and iodide (1.68 × 10^7^ m^−1^ s^−1^) are excellent two-electron donors of hsPxd01-con4 compound I and thus effectively compete with bromide. Reduction of compound I to the ferric resting state mediated by bromide (5.6 × 10^6^ m^−1^ s^−1^) is about 3-times slower compared with I^−^ and SCN^−^. Importantly, chloride (even at physiological concentrations, > 100 mm) could not mediate Reaction 5. Moreover, in the presence of chloride only compound I decayed to compound I* according to Reaction 2, whereas in the presence of 100 mm Cl^−^ and micromolar bromide Reaction 5 was followed. This data fit with (i) the observation that chloride did not support cross-link formation whereas addition of micromolar Br^−^ rescued sulfilimine formation (2) and with (ii) the fact that *E*′^0^ [Fe(III)/Fe(II)] of hsPxd01-con4 is significantly less positive compared with that of MPO, which is the only known human enzyme that is able to oxidize chloride at reasonable rate at neutral pH. This enzymatic property is closely related to the MPO-specific covalent heme-protein sulfonium ion linkage which does not exist in hsPxd01 (3, 4, 9). Nevertheless, hsPxd01-con4 outperforms the reactivity of LPO and MPO toward bromide at neutral pH (Table 1).

Typical normal human serum concentrations of bromide are in the range 10–100 μm, and the Br^−^ level is maintained via diet and renal excretion (33). *In vitro* studies on sulfilimine formation together with modeling of the cross-linking reaction clearly demonstrated that hypobromous acid is responsible for the formation of a bromosulfonium-ion intermediate that energetically selects for sulfilimine formation (2). Based on the demonstration that (i) bromine deficiency leads to physiological dysfunction, (ii) that repletion of the element reverses dysfunction, and that (iii) biochemical data can explain the physiological function, bromine has to be considered to be an essential trace element in animals (2). Its oxidation by hsPxd01 according to Reaction 5 provides the basis for the biosynthesis of sulfilimine cross-linked collagen IV scaffolds that are central to the formation and function of basement membranes in animals (1, 2).

However, because bromine has not been considered as an essential trace element until recently, systematic investigations on its replacement have not been pursued in various disease states associated with bromide deficiency. Functional Br^−^ deficiency may occur in smokers despite normal Br^−^ levels because of elevated levels of serum SCN^−^. Normally, the level of thiocyanate in blood plasma varies in individuals from 20 to 120 μm depending on their diet but can be significantly increased in smokers (34). Under these conditions, SCN^−^ would be the preferred electron donor for compound I, and reinforcement of collagen IV scaffolds with sulfilimine cross-links may be substantially reduced. Indeed, smoking has been associated with changes in the architecture of basement membranes (35). Oxidation of SCN^−^ generates hypothiocyanate, which is a milder oxidant than hypobromous acid and reacts with thiol residues mainly (36, 37) but cannot mediate the formation of sulfilimine cross-links. Because iodide levels are below micromolar concentrations in blood plasma, I^−^ will not compete with Br^−^ for hsPxd01 compound I.

Summing up, we were able to recombinantly produce a fully functional truncated human peroxidasin 1 variant with post-translationally modified and cross-linked heme. It allowed for the first time the determination of apparent bimolecular rate constants of all relevant redox steps of the physiologically relevant halogenation cycle, *i.e.* the H_2_O_2_-mediated compound I formation followed by two-electron reduction of compound I by bromide, iodide, and thiocyanate. Besides EPO and MPO (Table 1), human peroxidasin 1 is shown to be the most effective generator of hypobromous acid in the human body.

## Experimental Procedures

### 

#### 

##### Materials

Bovine lactoperoxidase (L2005), sodium chloride, potassium thiocyanate, sodium cyanide, and hydrogen peroxide (30% solution) were purchased from Sigma. The concentration of hydrogen peroxide was determined at 240 nm using the molar extinction coefficient of 39.4 m^−1^ cm^−1^ (38). Potassium bromide and potassium iodide were obtained from Merck. All other chemicals, if not stated otherwise, were purchased from Sigma at the highest grade available. H_2_O_2_ solutions and potassium iodide were prepared fresh before use.

##### Cloning of hsPxd01-con4

Cloning, transient transfection, and expression of hsPxd01-con4 was described previously (5). The work presented here was performed with the N-terminal polyhistidine tag version of hsPxd01-con4, resulting in a translation product of 1069 amino acid residues (Pro^246^–Asp^1314^). All amino acid residue numberings refer to the full-length hsPxd01, including the signal peptide.

##### Purification of hsPxd01-con4

The cell supernatant was harvested and filtrated with a 0.45-μm PVDF membrane (Durapore) and stored at −30 °C until further processing. After thawing, the supernatant was stirred for 48 h at 4 °C before the volume was decreased (∼25 times), and the cell culture medium was replaced with 100 mm phosphate buffer, pH 7.4, using a Millipore Labscale^TM^ TFF diafiltration system. 5 ml of His-Trap^TM^ FF columns (GE Healthcare) loaded with nickel chloride were used for the purification of hsPxd01-con4. The column was equilibrated with 100 mm phosphate buffer, pH 7.4, containing 1 m NaCl and 5 mm imidazole. The sample was adjusted to 1 m NaCl and 5 mm imidazole before loading, and the column was washed with equilibration buffer after sample loading. The protein was eluted by applying two consecutive gradients of 0–8% (2 ml/min, 10 min) and 8–70% (1 ml/min, 50 min) of 100 mm phosphate buffer, pH 7.4, containing 500 mm NaCl and 500 mm imidazole, respectively. Eluted fractions were analyzed by UV-visible spectroscopy, SDS-PAGE, and Western blotting following standard procedures (Penta·His Antibody, BSA-free from Qiagen; anti-mouse antibody, alkaline phosphatase-conjugated).

Enhanced chemiluminescence was used for the detection of covalent heme to protein linkages as described earlier (5). Fractions were pooled accordingly and concentrated in a 10-kDa molecular mass cutoff dialysis tubing (SnakeSkin^TM^, Thermo Fisher Scientific) by applying PEG (20 kDa) to the outside of the tubing. Subsequently, the sample was dialyzed against 100 mm phosphate buffer, pH 7.4, and stored at −30 °C.

##### Spectral Characterization of hsPxd01-con4

The extinction coefficients of hsPxd01-con4 were determined to be 147,500 m^−1^ cm^−1^ at 280 nm and 101,400 m^−1^ cm^−1^ at the heme Soret peak, resulting in a theoretical purity number of 0.7 (ϵ_412 nm_/ϵ_280 nm_). The average purity number obtained by metal affinity chromatography was 0.45–0.55 indicating a 65–80% heme occupancy. Specified hsPxd01-con4 concentrations were always related to heme concentrations.

##### Spectroelectrochemistry

The standard reduction potential (*E*′^0^) of the Fe(III)/Fe(II) couple of hsPxd01-con4 was determined as described previously (5). Briefly, the spectroelectrochemical titrations were performed using a homemade OTTLE (optically transparent thin layer spectroelectrochemical) cell. The three-electrode configuration consisted of a gold mini-grid working electrode (Buckbee-Mears), a saturated calomel (Hg_2_Cl_2_) microreference electrode (AMEL Electrochemistry), separated from the working solution by a Vycor set, and a platinum wire as counter-electrode (11, 21, 29). All potentials are referenced to the standard hydrogen electrode.

Experiments were performed with 5 μm hsPxd01-con4 in 100 mm phosphate buffer, pH 7.4, containing 100 mm NaCl, 30 μm methyl viologen, and 1 μm lumiflavin 3-acetate, methylene blue, phenazine methosulfate, and indigo disulfonate used as mediators at 25 °C. Nernst plots consisted of at least five points and were invariably linear with a slope consistent with a one-electron reduction process (11, 21, 29). The spectroelectrochemical experiments were performed three times, and the resulting *E*′^0^ values were found to be reproducible within ±6 mV.

##### Stopped-flow Spectroscopy

Pre-steady-state spectra were recorded with the stopped-flow apparatus SX.18MV (Applied Photophysics) connected to a diode array detector (DAD) with the first spectrum usually recorded 3 ms after mixing the reactants. The Pi-star-180 apparatus from Applied Photophysics was employed for all single wavelength measurements, and the first data point after mixing two solutions was typically recorded at 1 ms. The optical quartz cell had a volume of 20 μl and a path length of 10 mm. All reactions were followed at single wavelengths and additionally by using the DAD. Polychromatic data were analyzed with the Pro-Kineticist software from Applied Photophysics. Rate constants were determined by fitting single wavelength time traces with the Pro-Data Viewer software (Applied Photophysics). The conventional mode was applied to monitor the reaction of hsPxd01-con4 with hydrogen peroxide by following the decrease of absorbance at 412 nm and cyanide binding by monitoring the increase at 434 nm. All presented rate constants were measured using the sequential mixing mode due to the inherent instability of compound I. A delay time of 200 ms for the formation of compound I was employed.

All reactions with the exception of the pH profiles presented were performed in 100 mm phosphate buffer, pH 7.4, and at 25 °C. Citrate phosphate buffer was used for measurements from pH 4 to 5.5; phosphate buffer was employed for the pH range of 5.5–8, and carbonate buffer was used for pH 9 and 10. Three measurements were performed for each ligand (cyanide), oxidant (hydrogen peroxide), and electron donor (halides and thiocyanate) concentration, respectively. The mean of the first-order rate constants, *k*_obs_, was used to calculate the apparent second-order rate constant that was obtained from the slope of the plot of the *k*_obs_ values *versus* the concentrations of the respective reactants.

## Author Contributions

P. G. F. and C. O. conceived and coordinated the study and wrote the paper. M. P. P. designed the constructs, performed and analyzed the experiments, and contributed to writing of the paper. R. S. K., I. S., E. E., B. S., and M. S. provided technical assistance and produced and purified the recombinant proteins. M. B. and G. B. performed the spectroelectrochemical experiments, and S. H. probed the homogeneity and conformational stability of the constructs.

## Footnotes

3 The abbreviations used are:

hsPxd01: human peroxidasin 1
hsPxd01-con4: construct 4 of human peroxidasin 1
POX: peroxidase domain
LRR: leucine-rich repeat domain
VWC: von Willebrand factor C
LPO: lactoperoxidase
EPO: eosinophil peroxidase
MPO: myeloperoxidase
CT: charge transfer
DAD: diode array detector.
